# Fluorescent Copper Nanomaterials for Sensing NO_2_^−^ and Temperature

**DOI:** 10.3389/fchem.2021.805205

**Published:** 2022-01-25

**Authors:** Ning Wang, Lu Ga, Jun Ai, Yong Wang

**Affiliations:** ^1^ Inner Mongolian Key Laboratory of Environmental Chemistry, College of Chemistry and Environmental Science, Inner Mongolia Normal University, Hohhot, China; ^2^ School of Pharmacy, Inner Mongolia Medical University, Hohhot, China; ^3^ College of Geographical Science, Inner Mongolia Normal University, Hohhot, China

**Keywords:** CuNPs, nitrite, temperature detection, nanomaterial, fluorescence

## Abstract

In this work, highly fluorescent copper nanomaterials were synthesized by using ascorbic acid as a ligand. The excitation wavelength of copper nanomaterials is 367 nm, and the emission wavelength is 420 nm. The size range is 5–6 nm. Nitrite can selectively quench the fluorescence of copper nanomaterials. Therefore, copper nanomaterials can be used to selectively detect nitrite ions. The linear equation is F = −32.94 c (NO_2_^−^) + 8,455, and the correlation coefficient is 0.9435. At the same time, we found that the fluorescence intensity of copper nanomaterials has a good correlation with temperature (20–60°C), which shows that they have great potential in the application of nanothermometers.

## Introduction

Nitrite, as a food additive and soil raw material, widely exists in food and environment. But excessive nitrite in actual water will cause serious harm to public health and environment (Davis and Compton, 2000; Kroupova et al., 2008; Wang et al., 2009; Zhao and Li, 2020; Jahan et al., 2021). When the concentration of nitrite is higher than 4.5 mg/ml (Iii and Finke, 1999), it will damage the nervous system, spleen, and kidney of human beings and even leads to a high risk of cancer. Therefore, accurate detection of nitrite in drinking water sources, wastewater treatment systems, the food industry, and environment is of great significance.

In recent years, copper nanoparticles with high fluorescence have been widely used in biomedicine (Zhang et al., 2018), bioimaging (Satyvaldiev et al., 2018), environmental detection (Zhong et al., 2015; Li et al., 2018), catalysis (Wang et al., 2019), and other fields due to their excellent photoelectric properties, great biocompatibility, good stability, and low toxicity. Wang’s group reported that copper nanoclusters were synthesized using glutathione as stabilizer and ascorbic acid as reductant, and they detected nitrite ions by utilizing the quenching fluorescence effect (Zhou et al., 2017). The method has been successfully applied to the detection of nitrite ions in actual water samples. Li’s group reported that copper nanomaterials were synthesized using cysteine (Li et al., 2015; Ashraf et al., 2019; Aziz et al., 2019; Ashraf et al., 2020; Ashraf et al., 2021; Iftikhar et al., 2021). Based on the aggregation induction (AIE) of copper nanoclusters induced by sulfur ions, they established a fluorescence analysis method for the determination of hydrogen sulfide. It was applied to the detection of practical samples. Current analytical challenges faced by researchers in carrying out nitrite detection and temperature sensing are that the methods used are not suitable for online monitoring and in-site detection as they require fine instruments and complicated sample pretreatment. Therefore, fluorescence sensing has emerged as a tempting analytical method, as it does not require expensive instruments and involves a simple pretreatment procedure. Herein, copper nanomaterials were selected because the synthesis cost of copper nanomaterials is low and the fluorescence signal is good.

In this study, highly fluorescent copper nanomaterials were synthesized with ascorbic acid as a protective and reducing agent. Nitrite ions can effectively quench the fluorescence of copper nanomaterials and realize the detection of nitrite ions. As the synthesized copper nanomaterials have good temperature sensitivity, they can also be used as temperature sensors. The fluorescence intensity of copper nanomaterials is linear at different temperatures. Therefore, the synthesized copper nanomaterials have great potential in ion sensing, detection, and drug delivery.

## Instrument, Reagent, and Experimental Section

### Instrumentation

Fluorescence spectrogram was obtained by using an F-4600 fluorescence spectrophotometer (Hitachi High Tech. Company). Transmission electron microscope (TEM) signals were obtained by a JEOL-2100F transmission electron microscope (Japan) operating at a voltage of 200 kV. TEM sample characterization was carried out by dropping the sample in a carbon-coated copper mesh dispersion solution and drying at room temperature. An ESCALAB-250-XI X-ray photoelectron spectroscopy (XPS) analyzer was used (Thermo Fischer Scientific).

### Chemical Reagent

Ascorbic acid (C_6_H_8_O_6_); Cu(NO_3_)_2_·3H_2_O; ZnSO_4_·7H_2_O; Pb(NO_3_)_2_ (analytically pure, Tianjin Wind Ship Chemical Reagent Technology Co., Ltd.); Bi(NO_3_)_3_·3H_2_O; AgNO_3_ (analytically pure, Beijing Fuchen Chemical Reagents Co., Ltd.); FeCl_2_·4H_2_O (analytically pure, Beijing Shle Chemical Plant); MnSO_4_·H_2_O (analytically pure, Beijing Chaoyang District Chemical Four Plant); Ni(NO_3_)_2_·6H_2_O (analytically pure, Beijing 5671 Chemical Plant); Al(NO_3_)_3_·9H_2_O (analytically pure, Tian Jin Bo Di Chemical Co., Ltd.); sodium citrate (analytically pure, Tian Jin Public and Private Joint Chemical Reagent First Plant); Cd(NO_3_)_2_ (analytically pure, Beijing Chemical Co., Ltd.); CoCl_2_•6H_2_O (analytically pure, Shang Hai Public and Private Joint Venture Factory); NaF (Beijing Public and Private Chemical Plant); NaCl, Na_2_S_2_O_3_·5H_2_O, and KCl (Tian Jin North Union Fine Chemicals Development Co., Ltd.); AgNO_3_ (Beijing Fuzhou Chemical Reagents Co., Ltd.); NaNO_2_ (Tianjin Public and Private Chemical Reagents First Plant); NaBr and NH_4_Cl (Tian Jin People Chemical Plant); Na_2_SiO_3_.9H_2_O (Beijing Yizhuang Middle School Chemical Plant); and water used in all experiments is ultra-pure water with a resistance of 18.25 MΩ.

### Synthesis of CuNPs

The specific method for synthesizing copper nanomaterials is as follows: first, 0.5 ml of 1 mmol/L copper nitrate solution and 3 ml of 10 mmol/L ascorbic acid solution were mixed and stirred for 20 min. Then 10 mmol/L sodium hydroxide solution was used to adjust the pH of the mixed solution to 6. After that, the solution was transferred to a 60°C constant temperature water bath for 5 h. Finally, after centrifugation, the supernatant of copper nanomaterials was preserved at 4°C for later use.

### Detection of NO_2_^−^

Of the prepared copper nanoparticles, 450 ml was placed in 1.5-ml centrifuge tubes, and then 50 ml of nitrite solution was added at different concentrations to the centrifuge tubes. The fluorescence intensity was measured at 365 nm after 40 min reaction at room temperature.

### Copper Nanomaterials for Temperature Sensing

The copper nanomaterial solution was placed in 1.5-ml centrifuge tubes. The tubes were heated at different temperatures for 20 min, and then the fluorescence intensities of the solutions were measured with a fluorescence spectrophotometer.

## Results and Discussion

### Characterization of Copper Nanomaterials

**FIGURE 1:**
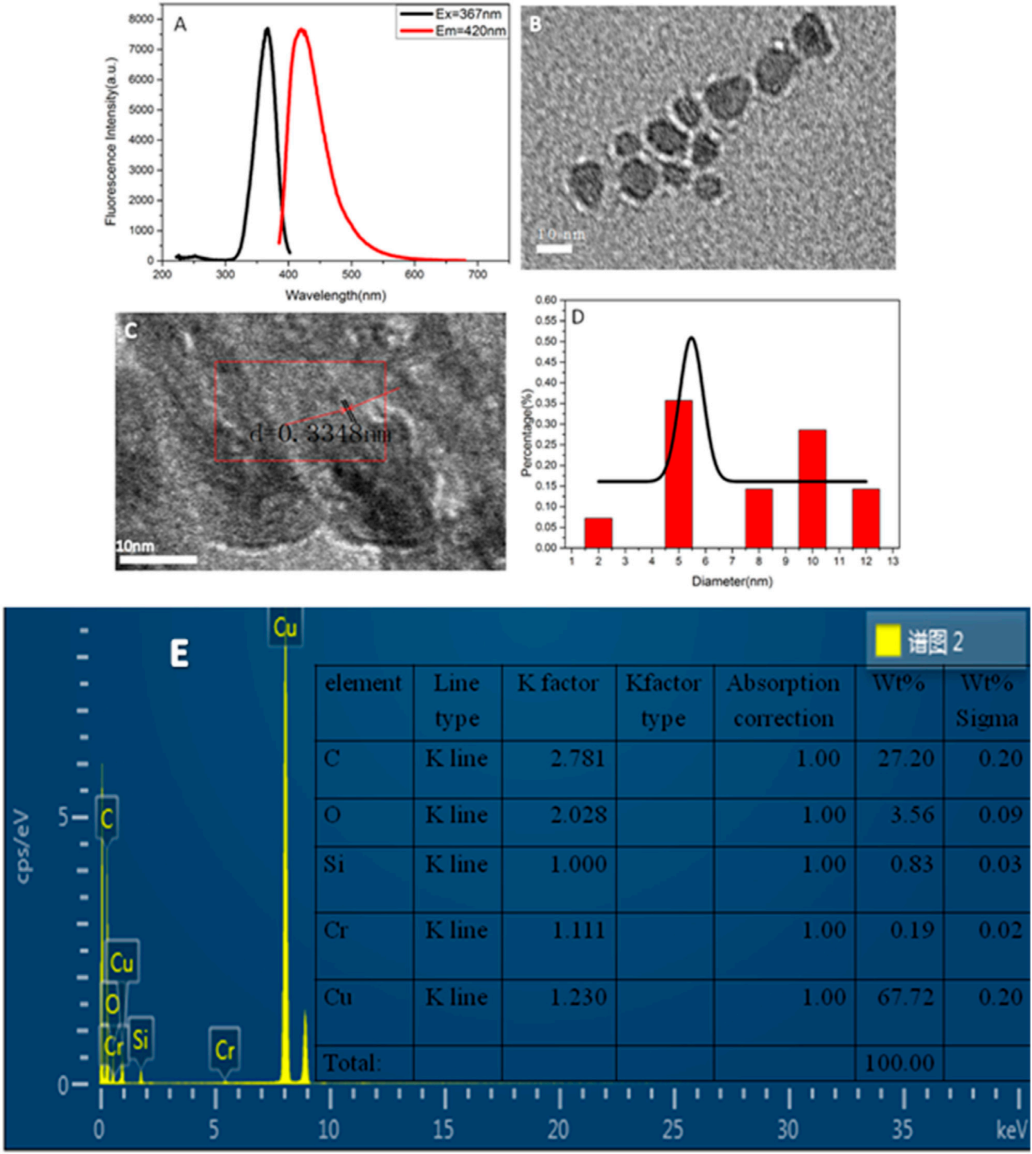
**(A)** Fluorescence spectra of copper nanomaterials; **(B)** TEM image of copper nanomaterials; **(C)** HRTEM image of copper nanomaterials; **(D)** size distribution histogram of copper nanomaterials; **(E)** ED image of CuNPs.

Figure 1A shows the excitation and emission spectra of copper nanomaterials. It demonstrates that the synthesized copper nanomaterials have a strong fluorescence signal, and the maximum emission wavelength is 420 nm. The copper nanomaterials were dropped on the nickel mesh of the carbon support. The samples were dried and tested by TEM and energy dispersive spectroscopy (EDS). The morphology of the prepared copper nanomaterials was characterized by TEM (Figure 1B). As shown in Figure 1B, the copper nanomaterials are uniformly dispersed and have a smaller particle size. Figure 1C presents a high-resolution transmission electron microscopy (HRTEM) image. The crystal lattice of d = 0.3348 nm corresponding to the face-centered cubic structure (110) of copper can be clearly seen. Figure 1D indicates that the particle size distribution of the copper nanomaterials is uniform, and the average diameter of the copper nanomaterials is in the range of 5.0 ± 0.1 nm. The EDS image and the elemental contents in the copper nanomaterials are presented in Figure 1E. The percentage of copper was 67.72%. The results indicated that the synthesized copper nanomaterials have the characteristics of good fluorescence, uniform dispersion, and small particle size, which indicates that highlyfluorescent copper nanomaterials have been successfully synthesized.

**FIGURE 2:**
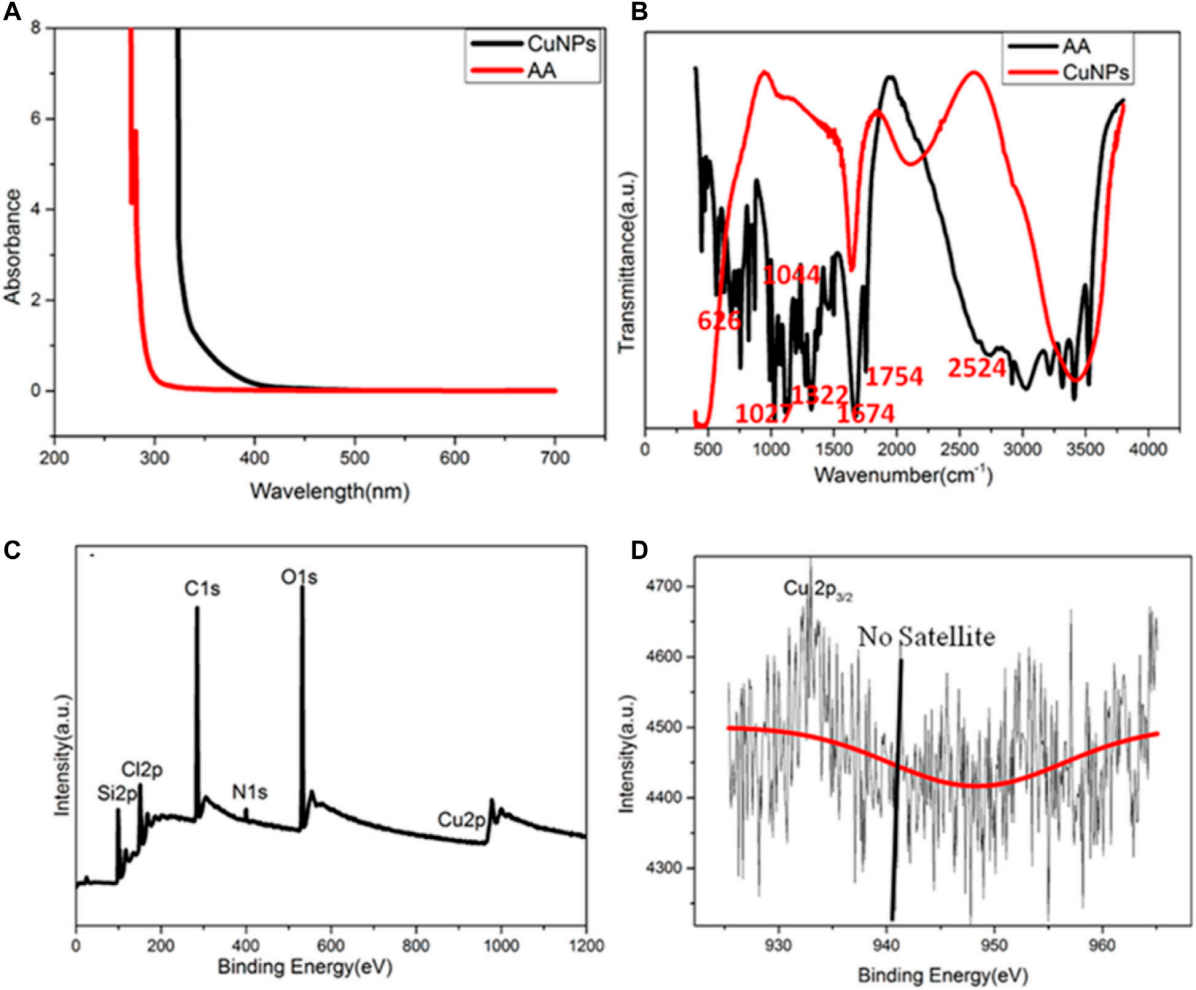
**(A)** UV–vis spectra of CuNPs and AA; **(B)** FT-IR spectra of CuNPs and AA; **(C)** XPS of CuNPs thus obtained; **(D)** amplified XPS of Cu 2p electrons.

The chemical and surface properties of the copper nanoparticles were analyzed by ultraviolet-visible (UV) spectroscopy and Fourier transform infrared (FT-IR) spectroscopy. Figure 2A shows the ultraviolet absorption spectra of CuNPs and ascorbic acid. There is no obvious absorption peak of ascorbic acid but a small ultraviolet absorption peak of CuNPs near 340 nm, which corresponds to the fluorescence excitation wavelength of copper nanomaterials. Figure 2B exhibits the infrared spectra of the copper nanomaterials and ascorbic acid, which can broaden the characteristic peaks of CuNPs. The shrinkage vibration peak of 2,524 cm^−1^ belonging to -OH has disappeared in the copper nanomaterials, which proves that glutathione molecules bond to the surface of copper nanomaterials through Cu-O. The characteristic peaks of ascorbic acid did not appear, which proves that the ligand exchange was complete.

XPS was used to characterize the valence state of copper in CuNPs. The full spectrum indicated that CuNPs consist of C, O, N, Si, and Cu (Figure 2C). Figure 2D shows that no satellite peaks appear, which means that no Cu^2+^ exists. The strong peak of 932.3 eV belongs to zero-valent copper’s 2p3/2, and this is consistent with previous literature reports (Koski et al., 2012). However, it should be noted that the binding energy of 2p3/2 of Cu(0) is similar to that of Cu(I). Due to the charge transfer of the Cu-O bond, the valence state of Cu nanomaterials may be between 0 and +1.

### Optimization of Experimental Synthesis Conditions

**FIGURE 3:**
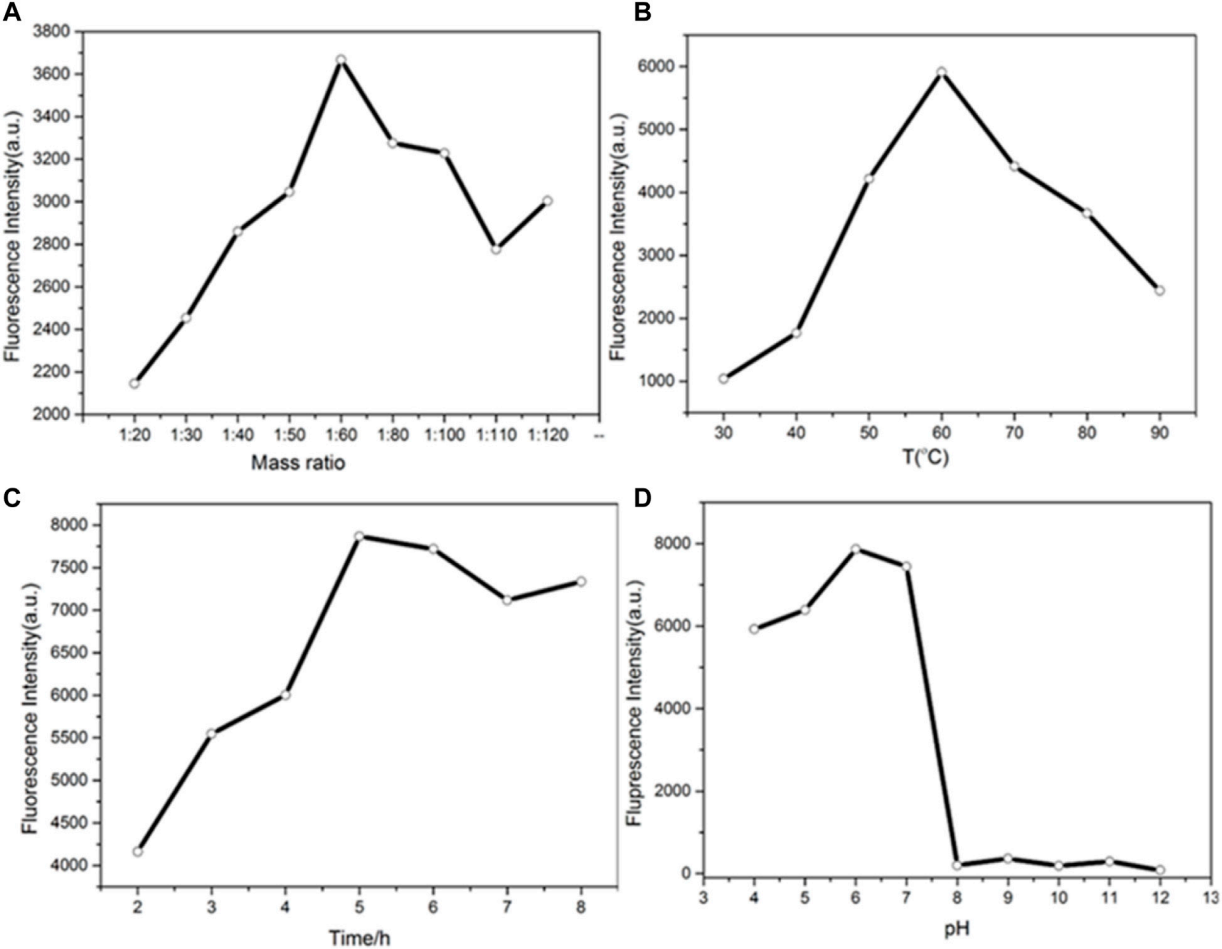
**(A)** Determination of the mass ratio of the synthesized CuNPs; **(B)** temperature of the synthesized CuNPs; **(C)** time of CuNP synthesis; **(D)** pH of CuNP synthesis.

The synthesis time, temperature, molar ratio, and pH were optimized. By adjusting the molar ratio of copper nitrate to ascorbic acid, the appropriate drug dosage for the synthesis of copper nanomaterials was determined. As shown in Figure 3A, when the molar ratio of copper nitrate to ascorbic acid is 1:60, the fluorescence intensity of copper nanomaterials is higher. Therefore, the best molar ratio is 1:60. Figure 3B shows the effect of synthesis temperature on fluorescence intensities. When the temperature was changed from 30 to 90°C, the fluorescence intensities of CuNPs first increased and then decreased. The fluorescence intensity of CuNPs was the highest at 60°C. Therefore, the optimal synthesis temperature of copper nanomaterials is 60°C. Figures 3C,D show the optimum time and pH data for the synthesis of copper nanomaterials. With the increase in time, the fluorescence intensity of copper nanomaterials increases. When the synthesis time is set to 5 h, the fluorescence intensity is the highest (Figure 3C). Therefore, the optimal synthesis time of copper nanomaterials is 5 h. As shown in Figure 3D, the fluorescence intensity of copper nanomaterials is the highest at pH 6. Therefore, we chose pH 6 as the best condition. Here, we synthesized CuNPs under the above optimum conditions.

### Stability of CuNPs

**FIGURE 4:**
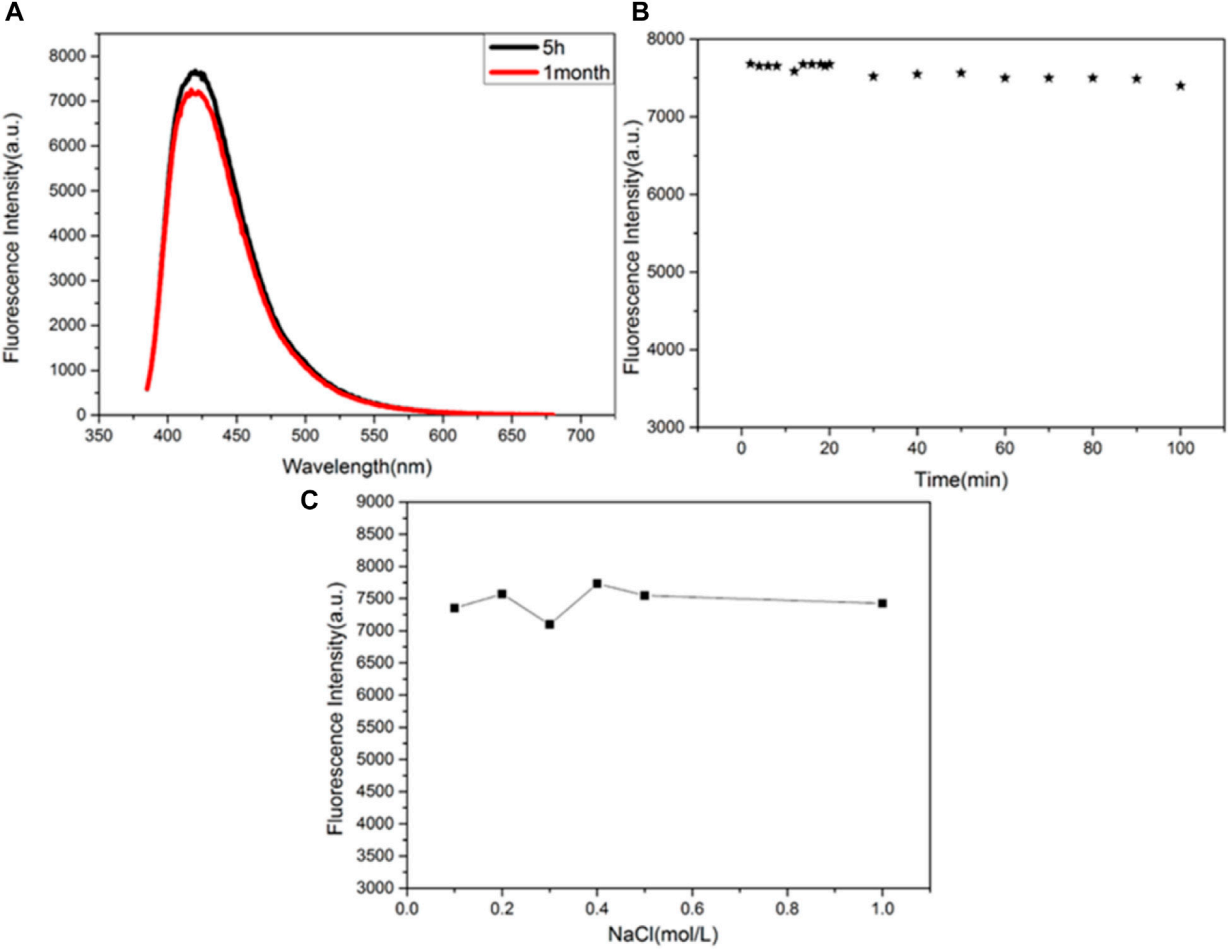
**(A)** Storage time; **(B)** irradiation time; **(C)** effects of the concentration of NaCl solution on the fluorescence intensity of the CuNPs.

The stability of the synthesized CuNPs was evaluated by comparing the fluorescence intensities of CuNPs stored for different periods and at different conditions (Figure 4). Figure 4A shows that the fluorescence intensity of the synthesized CuNPs measured at 5 h and 1 month after storage remains basically unchanged. It is concluded that CuNPs can be preserved for 1 month at 4°C. The fluorescence intensities of CuNPs were almost unchanged when the CuNPs were exposed to a xenon lamp for 100 min (Figure 4B). Figure 4C depicts the effect of different concentrations of NaCl solutions on the stability of CuNPs. It was found that the fluorescence intensity of copper nanomaterials was independent of the concentration of NaCl solution (up to 1 mol/L). These results indicate that the nanomaterials have good storage and fluorescence stability.

### Selectivity

**FIGURE 5:**
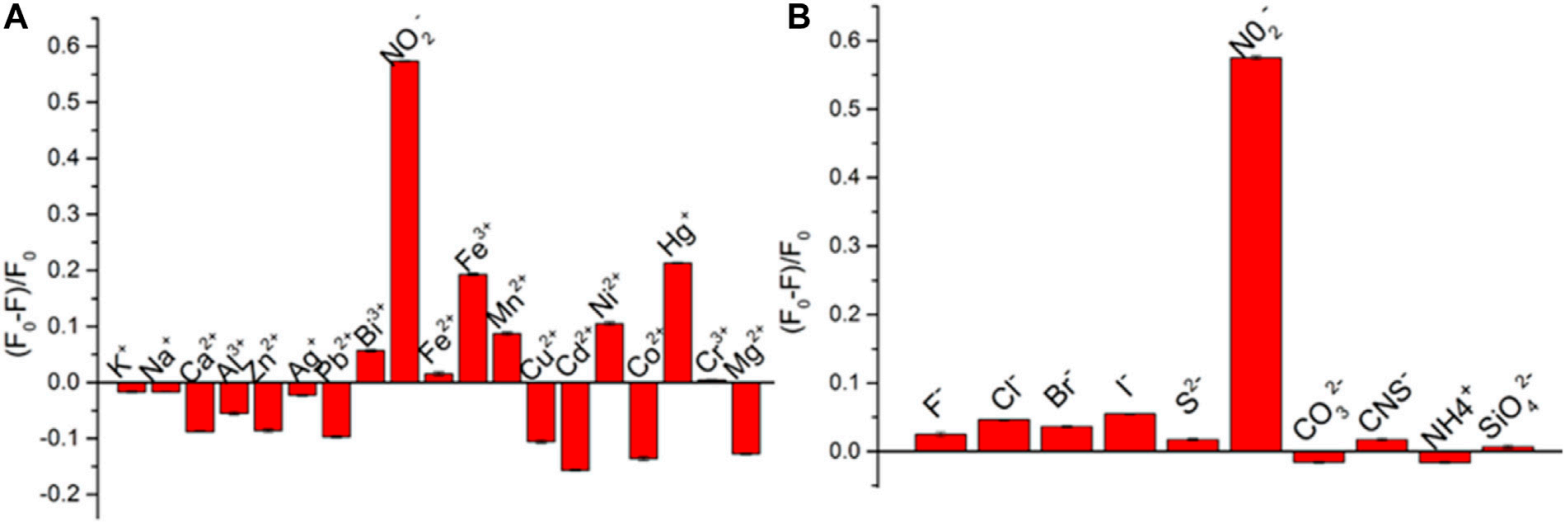
Selectivity of CuNPs toward I^−^ sensing over other common metal ions **(A)** and anions **(B)**. The concentration of all metal ions is 100 μmol/L.

To further investigate the selectivity of this method, contrast experiments were performed using 18 metal cations (K^+^, Na^+^, Ca^2+^, Hg^+^, Cr^3+^, Fe^3+^, Pb^2+^, and Bi^3+^) and 10 inorganic anions (F^−^, Cl^−^, Br^−^, I^−^, S^2−^, and NO_2_^−^) as the interference for detection of nitrite ions at the concentration of interference are 100 μmol/L. As shown in Figure 5, only nitrite ions show a strong quenching effect on the fluorescence of CuNPs. While Cd^2+^, Fe^3+^, and Hg^+^ have little effect on the fluorescence of CuNPs, other ions have no obvious effect on it. All these results indicate that this method possesses good selectivity for nitrite ion detection.

### Detection of NO_2_^−^

**FIGURE 6:**
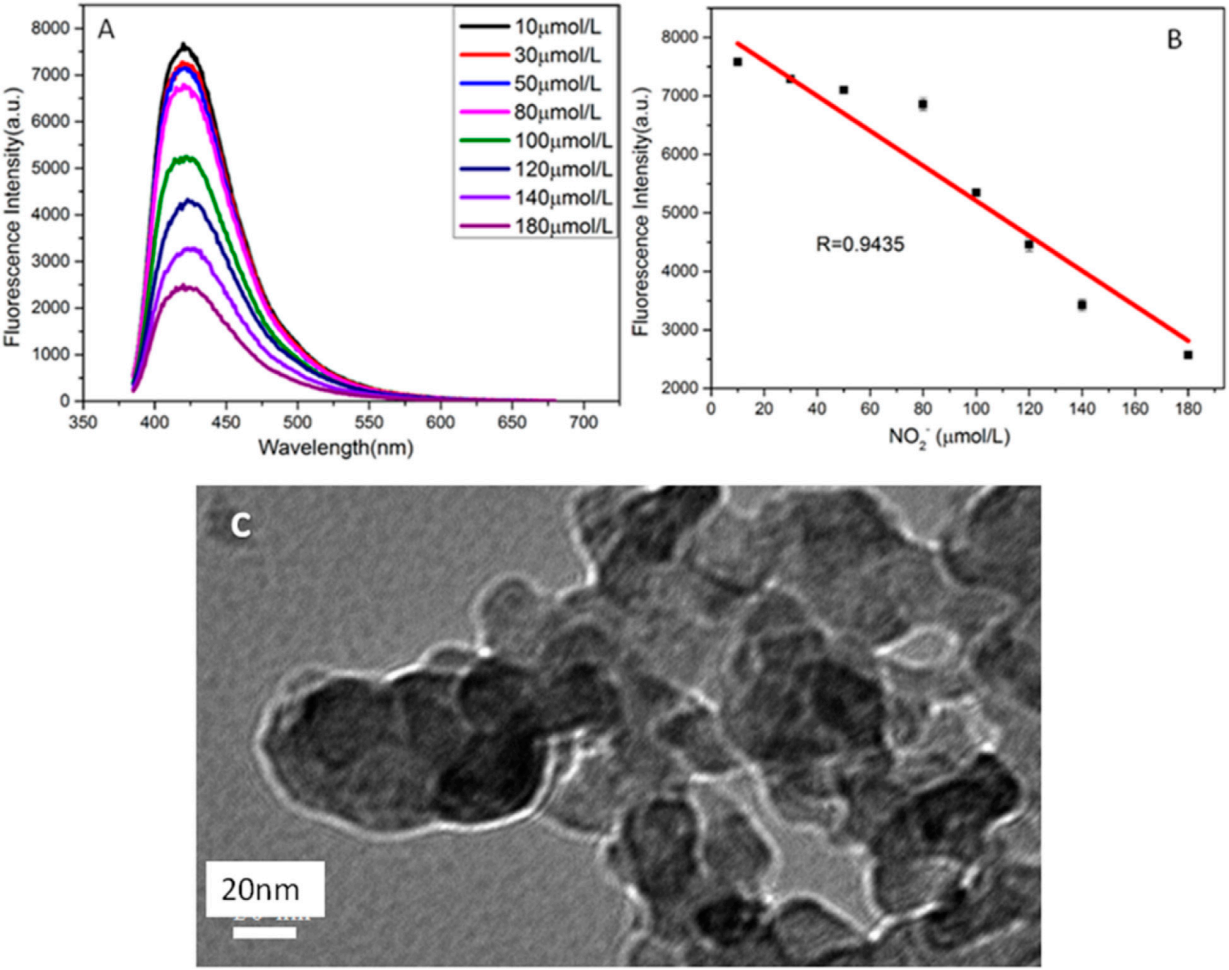
**(A)** Fluorescence spectra of CuNPs obtained with different concentrations of NO_2_^−^ (from top to bottom: 10, 30, 50, 80, 100, 120, 1,400, and 180 μmol/L, respectively); **(B)** relationship between F and the concentration of NO_2_^−^; **(C)** TEM of copper nanomaterials after adding NO_2_^−^.

Based on the quenching effect of nitrite ions on the fluorescence of CuNPs, we established a fluorescence quenching sensor for accurate detection of nitrite ions. Figure 6A indicates that the fluorescence intensities of copper nanomaterials decrease with the increasing concentration of nitrite ions. Figure 6B shows a good linear relationship between the quenching fluorescence intensities of CuNPs and nitrite concentration in the range of 10 *μ* M–180 μ M. The linear equation is F = −32.94 c (NO_2_^−^) + 8,455, and the correlation coefficient is 0.9435. Figure 6C shows the TEM image of CuNPs after adding NO_2_^−^. It demonstrates that the fluorescence quenching is caused by the aggregation of nanomaterials because of the interaction between the surface ligand (ascorbic acid) of CuNPs and NO_2_^−^. The results are basically consistent with previous reports. Nitrite is a kind of food additive and is used for its coloring and antiseptic properties. It is widely used in cooked meat and canned animal food and is also used as an enema agent. Nitrite is not only a carcinogen but can also cause food poisoning when ingested at 0.2–0.5 g and can cause death at 3 g. Accurate detection of the nitrite content in food online is a key problem.

### Temperature Sensing

**FIGURE 7:**
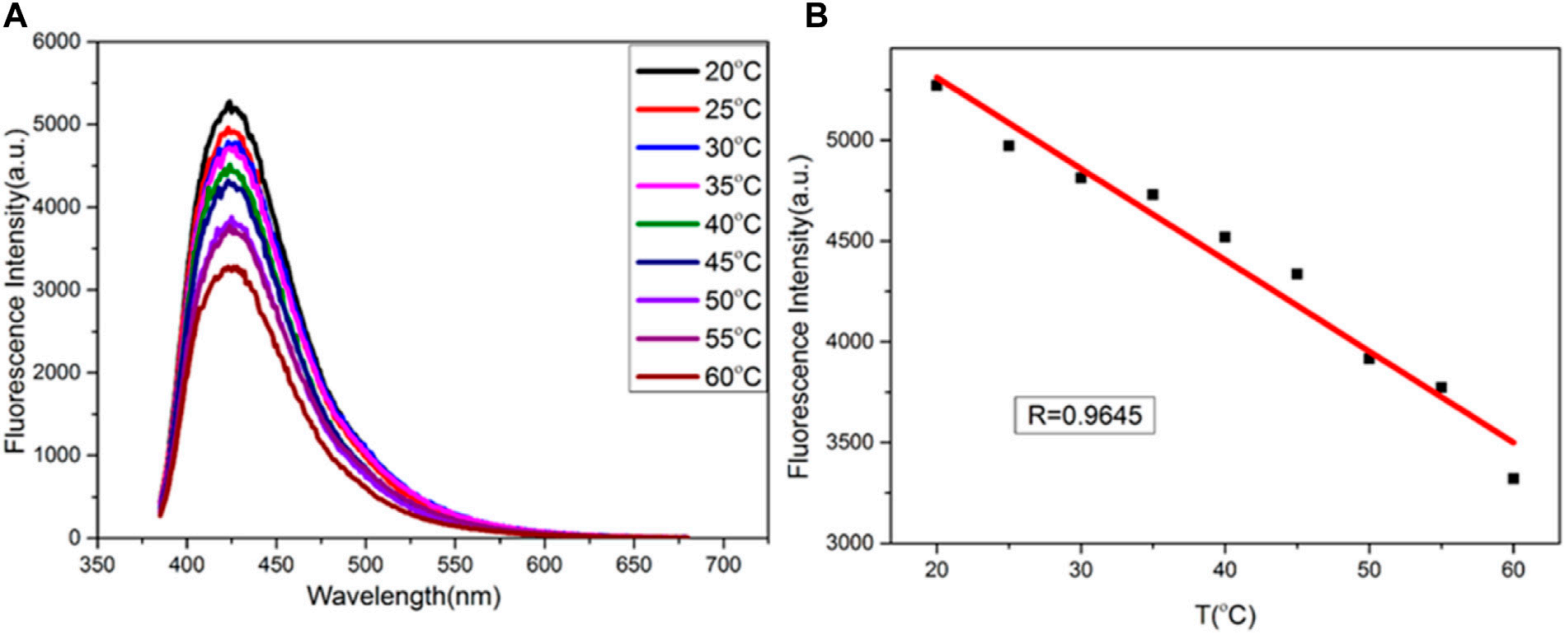
**(A)** Fluorescence spectra of CuNPs obtained at different temperatures (from top to bottom: 20, 25, 30, 35, 40, 45, 50, 55, and 60°C, respectively); **(B)** relationship between F and temperature.

A temperature-sensitive sensor based on copper nanomaterials was studied. As shown in Figure 7A, the fluorescence intensity of copper nanomaterials varies with temperature, from 20°C to 60°C. There is a good linear relationship between fluorescence intensity and temperature. The calibration equation is F = − 42.62T + 7,200, and the correlation coefficient is 0.9645 (as shown in Figure 7B). Therefore, the synthesized copper nanomaterial shows great potential in the application of nanothermometers.

## Conclusion

In summary, we synthesized a new kind of copper nanomaterial using ascorbic acid as both protectant and reductant. Nitrite ions can selectively quench the fluorescence of copper nanomaterials. Based on the fluorescence quenching mechanism, a sensor for detecting nitrite ions was established. We also found that there is a linear relationship between fluorescence signal and temperature in the temperature range of 20–60°C. Therefore, our new copper nanomaterials show great potential in the application of nanothermometers.

## Data Availability

The original contributions presented in the study are included in the article/Supplementary Material; further inquiries can be directed to the corresponding author.
